# Enhancing Biological Control of Maize Seedling-Stage Pests Through Combined Application of *Cnidium monnieri* and Endophytes

**DOI:** 10.3390/insects17080872

**Published:** 2026-08-21

**Authors:** Yingying Song, Lili Li, Kelin Cheng, Shuguang Wang, Ritao Qu, Hongying Cui, Wenxiu Guo, Suhong Lv, Ruohan Ma, Xingyuan Men

**Affiliations:** 1 Jinan Engineering Research Center of Ecological Agricultural Products R&D and Industrialization, Shandong Key Laboratory for Green Prevention and Control of Agricultural Pests, Key Laboratory of Biological Control of Pests, Ministry of Agriculture and Rural Affairs, Institute of Plant Protection, Shandong Academy of Agricultural Sciences, Jinan 250100, China; 2 College of Plant Health and Medicine, Qingdao Agricultural University, Qingdao 266109, China; 3 Yantai Agricultural Technology Promotion Center, Yantai 264000, China

**Keywords:** maize pests, *Cnidium monnieri*, endophytic fungi, conservation biological control, endophyte-associated pest suppression, sustainable pest management

## Abstract

Agricultural intensification has weakened natural pest regulation by reducing habitat resources for natural enemies and limiting plant-mediated resistance. Here, we conducted field experiments during 2024–2025 to evaluate the combined effects of *Cnidium monnieri* field-margin planting and maize seeds coated with the endophytic fungal strains YC (*Beauveria bassiana*) and PL (*Purpureocillium lilacinum*) on predator conservation, pest suppression, and biological control in maize agroecosystems. Compared with natural grass margins, *C. monnieri* significantly increased predator abundance, species richness, and Shannon diversity, particularly of ladybirds, minute pirate bugs, and spiders (*p* < 0.05). Gut-content analysis detected *C. monnieri* DNA in ladybirds collected 10–40 m from field margins, confirming predator resource use and spillover into maize fields. Importantly, endophytic fungus-coated seeds did not reduce predator abundance or diversity. Moreover, combining *C. monnieri* with endophytic fungi enhanced pest suppression and achieved control efficacy comparable to imidacloprid (*p* > 0.05). These findings demonstrate that integrating functional plants with endophytic fungi simultaneously strengthens top-down biological control and endophyte-associated bottom-up pest suppression, providing an effective and environmentally sustainable strategy for pest management in maize.

## Simple Summary

Agricultural intensification and climate change are increasing pest risks by disrupting ecological processes that naturally regulate herbivore populations. However, conventional insecticide-based management of maize pests often reduces beneficial arthropods and undermines ecosystem stability. Here, we developed an integrated pest management strategy by combining the functional plant *Cnidium monnieri* with endophytic fungus-coated maize seeds to simultaneously enhance natural enemy conservation and endophyte-associated pest suppression. Field experiments conducted over two consecutive years showed that *C. monnieri* planted at field margins substantially increased predator abundance and diversity and promoted predator spillover into adjacent maize fields, as confirmed by gut-content analysis of ladybirds. Endophytic fungus seed coatings further enhanced pest suppression without negatively affecting predator communities. The combined strategy achieved comparable pest control to imidacloprid treatment. Our findings demonstrate that integrating habitat-based and endophyte-associated defenses can provide a sustainable approach for improving biological control and reducing reliance on chemical insecticides in agricultural ecosystems.

## 1. Introduction

Climate warming and increasing extreme weather events are intensifying agricultural pest risks by expanding pest distributions, accelerating development rates, and increasing the number of generations per year [1,2,3]. In this context, agricultural intensification exerts a dual influence on pest dynamics [4,5,6]. On one hand, simplified cropping systems and the reduction of non-crop habitats limit the availability of resources for natural enemies, thereby weakening predator-mediated biological control [7,8]. On the other hand, intensification undermines the long-term stability of crop resistance by reducing genetic diversity, simplifying agroecosystems, and facilitating rapid pest adaptation [1,9]. These combined pressures frequently lead to pest outbreaks, posing significant threats to crop yield and sustainability [10,11]. Therefore, developing integrated pest management strategies that simultaneously enhance natural enemy recruitment and strengthen plant resistance is essential for achieving stable and sustainable pest control under intensive agricultural systems.

Functional plants can improve conservation biological control by providing nectar, pollen, shelter, and alternative prey, thereby increasing the survival, reproduction, and foraging ability of predators and parasitoids [12,13]. Flowering strips and insectary plants further enhance pest suppression by improving natural enemy dispersal, habitat connectivity, and their ability to colonize crop fields, ultimately strengthening landscape-level pest regulation [14,15,16]. Among these, *Cnidium monnieri* (L.) Cusson has gained attention due to its prolonged flowering period, abundant floral resources, and strong attractiveness to key predators such as *Harmonia axyridis* and *Propylaea japonica*, with floral volatiles mediating their attraction and enhancing biological control potential [17,18]. In China, *C. monnieri* is widely established as a field-margin habitat plant, typically sown in autumn and flowering from May to July [19]. In wheat–maize rotation systems, its flowering period extends into mid-July and substantially overlaps with the maize seedling stage, a critical period when crops are highly vulnerable to early pest colonization. This phenological synchrony suggests that *C. monnieri* may provide ecological support for natural enemies during early maize growth; however, whether and how *C. monnieri*-mediated habitat manipulation influences natural enemy communities and pest populations in maize systems remains insufficiently understood.

In addition, plant-associated endophytic microorganisms have attracted increasing attention due to their important roles in enhancing plant resistance to herbivorous insects and improving crop resilience [20,21]. Beneficial endophytes, including bacteria and fungi, can colonize internal plant tissues and activate plant defense pathways such as jasmonic acid (JA) and salicylic acid (SA) signaling, thereby inducing systemic resistance against a wide range of insect pests [22,23,24]. Seed coating with endophytes has been shown to reduce herbivore performance by decreasing feeding, survival, and reproduction [21,25,26].

Integrating habitat management with plant-mediated resistance may offer a promising approach for robust and sustainable pest control. Functional plants enhance top-down regulation by supporting predator populations, while endophytes reinforce bottom-up resistance within crops [18,27,28,29]. The combination of these strategies may achieve stronger pest suppression than either approach alone. However, most studies have been conducted under controlled laboratory or greenhouse conditions, and the ecological effects of integrating endophytes with habitat management practices, such as functional plants or flowering strips, remain largely unexplored in field environments.

Therefore, this study aimed to evaluate the combined effects of field-margin planting of *C. monnieri* and endophytic seed coating on predator communities and pest suppression during the maize seedling stage. We hypothesized that (1) *C. monnieri* planting would increase predator abundance and diversity in maize fields, (2) endophytic seed coating would reduce pest abundance in maize, and (3) their combination would provide enhanced pest suppression through complementary ecological mechanisms involving predator-mediated regulation and endophyte-associated pest suppression.

## 2. Materials and Methods

### 2.1. Endophytic Fungi and Maize Seed Coating

Insect-resistant endophytic fungal strains YC (*Beauveria bassiana*) and PL (*Purpureocillium lilacinum*) were isolated and screened in the previous study in our laboratory [27,30].

The maize variety used in this study was Zhengdan 958, purchased from Beijing Denong Seed Industry Co., Ltd., Beijing, China, with a total growth period of 95 days. Untreated maize seeds (without any coating) were used for endophytic fungal seed-coating treatments, while maize seeds coated with imidacloprid served as the conventional chemical control treatment.

For seed coating, each fungal preparation (4 × 10^10^ viable conidia g^−1^, 50 g per mu; corresponding to a theoretical inoculum dose of approximately 4.8 × 10^8^ viable conidia per seed based on the planting density (63,000 plants ha^−1^)) was mixed with an appropriate amount of methyl cellulose (as an adhesive; Shanghai Yuan Ye Biotechnology Co., Ltd., Shanghai, China) and talc powder (as a carrier; Guangxi Longsheng Huamei Calcite Development Co., Ltd., Guilin, China). The mixtures were then applied to maize seeds using a mechanical seed-coating machine (BY25C; Linyi Lingxin Machinery Equipment Co., Ltd., Linyi, China) to ensure uniform coating. Fungal coating did not affect seed viability, as germination rates exceeded 90% in all treatments and showed no significant differences from the untreated maize seeds.

### 2.2. Functional Plants

The functional plant used in this study was *C. monnieri*, a high-yielding variety with a stable genetic background that was developed by our research team over many years [31].

### 2.3. Experimental Design

**Figure 1:**
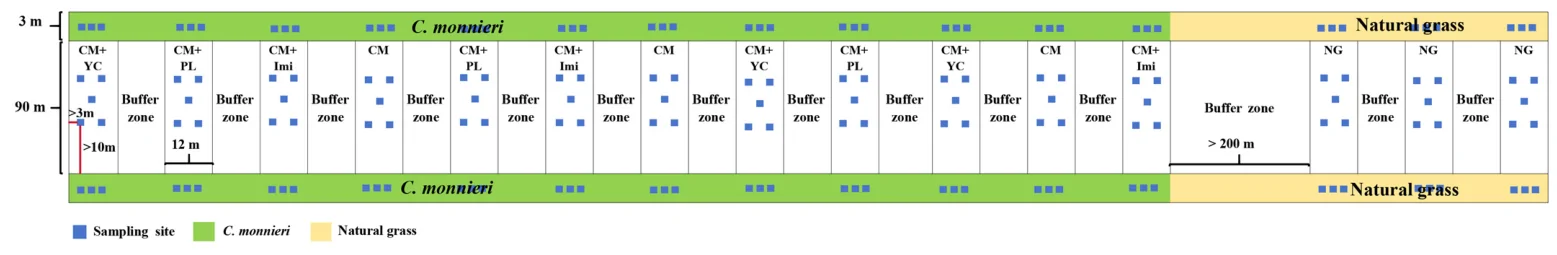
Schematic layout of the field experiment showing the arrangement of treatments under two vegetation backgrounds in 2024 and 2025. The experimental field was divided into two vegetation backgrounds, *Cnidium monnieri* and natural grass, each 3 m wide and located at opposite ends of the field. In the *C. monnieri* area, maize plots received endophytic fungus treatments (CM+YC and CM+PL), imidacloprid treatment (CM+Imi) and an untreated control (CM), while in the natural grass area, plots received an untreated control (NG). Each treatment was replicated three times in plots measuring 12 m × 90 m, arranged along the length of the field. Each plot was separated by buffer strips of 12 m. The two vegetation areas were separated by a distance greater than 200 m to reduce movement of natural enemies between backgrounds. Blue squares indicate sampling points established within each plot for monitoring insects and natural enemies. The red line indicates the measured distance between the sampling site and the adjacent boundary.

The experiment was conducted in 2024 and 2025 at the experimental fields located in Jiyang, China (36°58′ N, 116°58′ E). The experimental site was located on relatively flat agricultural land, and no obvious variation in topography was observed across the experimental area. The field was composed of two distinct vegetation backgrounds: *C. monnieri* plots and natural grass plots. The natural grass vegetation mainly consisted of *Cirsium setosum*, *Conyza canadensis*, *Humulus scandens*, *Digitaria sanguinalis*, *Eleusine indica*, *Setaria viridis*, and *Portulaca oleracea*. Each vegetation strip was 3 m wide and positioned at opposite ends of the field. Within the *C. monnieri* background, maize seeds were treated with two endophytic fungi (CM+YC and CM+PL), an imidacloprid treatment (CM+Imi), and an untreated control (CM). In contrast, the natural grass background included an untreated control (NG). Within each vegetation background, treatments were randomly assigned with three independent replicates, and each plot measured 12 m × 90 m. Each plot was oriented along the north–south direction. Adjacent plots were separated by isolation strips (buffer zones) of 12 m width to reduce potential interference among treatments. The two vegetation backgrounds were separated by a distance exceeding 200 m to limit the dispersal of natural enemies between them. Sampling points were established within each plot and distributed evenly for monitoring insect populations and their natural enemies (Figure 1).

*C. monnieri* was initially sown in early September 2023 at a seeding rate of 37.5 kg ha^−1^. The *C. monnieri* strips were managed under natural seed dispersal, allowing for perennial propagation; therefore, no re-sowing was required during 2024–2025. Maize was planted on 21 June 2024 and 18 June 2025 at a density of 63,000 plants ha^−1^. Throughout the experimental period, no insecticides were applied, and all other agronomic practices followed conventional local management.

### 2.4. Predator Populations, Pest Populations, and Maize Damage

Within each *C. monnieri* strip, three sampling points were randomly selected from areas with uniform plant growth. A 0.5 m × 0.5 m sampling frame was placed at each point, and all predators within the frame were recorded. For maize, populations of predators and pests were monitored using a five-point sampling method within each plot. Five sampling points were evenly distributed along the length of each plot and maintained at the same positions during the two experimental years. All sampling points were located at least 3 m from plot edges to minimize edge effects (Figure 1). At each point, at least 30 maize plants were randomly selected, and the numbers of predators and pests, as well as the number of damaged maize plants, were recorded. Surveys were conducted at the seedling stage on 12 July 2024 and 10 July 2025.

The maize damage rate was calculated as the proportion of damaged plants relative to the total number of surveyed plants:

$$\mathrm{Damage}\;\mathrm{rate}\;(\%)\;=\;\frac{\mathrm{Number}\;\mathrm{of}\;\mathrm{damaged}\;\mathrm{plants}}{\mathrm{Total}\;\mathrm{number}\;\mathrm{of}\;\mathrm{surveyed}\;\mathrm{plants}}\;\times \;100$$

The control efficacy was calculated as

$$\mathrm{Control}\;\mathrm{efficacy}\;(\%)=\frac{(\mathrm{Damage}\;\mathrm{rate}\;\mathrm{in}\;\mathrm{control}-\mathrm{Damage}\;\mathrm{rate}\;\mathrm{in}\;\mathrm{treatment})}{\mathrm{Damage}\;\mathrm{rate}\;\mathrm{in}\;\mathrm{control}}\;\times \;100$$

### 2.5. Detection of C. monnieri in Predators of Maize Fields

To investigate the role of *C. monnieri* in supporting predatory insects in maize fields, we examined the presence of *C. monnieri* DNA in their gut contents. We employed *C. monnieri*-specific primers (Forward: 5′-GCTCGCTAAGATGACGAGGATT-3′; Reverse: 5′-CACTCTCGGGTGGCCACT-3′) to detect plant material within ladybirds [32]. Gut contents of collected ladybirds were subjected to DNA extraction following the CTAB-based protocol described previously [32]. PCR amplification was performed in a 25 μL reaction mixture containing 1 μL template DNA, 12.5 μL Taq PCR Master Mix (2×), 1 μL forward primer (10 μM), 1 μL reverse primer (10 μM), and 9.5 μL sterile distilled water. The PCR program consisted of an initial denaturation at 94 °C for 10 min, followed by 35 cycles of denaturation at 94 °C for 30 s, annealing at 58 °C for 45 s, and extension at 72 °C for 30 s, with a final extension at 72 °C for 5 min. PCR products were separated on 1.5% agarose gels and visualized under UV illumination.

Sampling was conducted at maize field sites located 10, 20, 30, and 40 m from *C. monnieri* strips in 2025. At each distance, the gut contents of ladybirds were analyzed via PCR to assess the detectability of *C. monnieri*. At each distance, five ladybirds were collected per replicate, with three replicates per sampling point. As a negative control, ladybirds reared indoors without exposure to *C. monnieri* were included.

### 2.6. Monitoring Temperature and Humidity of C. monnieri and Maize Plants

To explore the potential drivers of predator movement from *C. monnieri* to maize fields, temperature and humidity were monitored in both plant systems using a digital temperature–humidity data logger (GSP-8G, Jiangsu Jingchuang Electric Co., Ltd., Suzhou, China). A dual-channel sensor was employed, with probes positioned near the central regions of maize leaves and *C. monnieri* leaves, respectively. Temperature and relative humidity were recorded at 5 min intervals, allowing continuous assessment of microclimatic conditions in the two plant canopies.

### 2.7. Data Analysis

Statistical analyses were performed using IBM SPSS Statistics 26.0 (IBM Corp., Armonk, NY, USA). Data were examined for normality and homogeneity of variances prior to analysis. Variables that did not satisfy these assumptions were log10- or arcsine-transformed before analysis, whereas untransformed means are presented in the figures. Two-way ANOVA was conducted to evaluate the effects of vegetation type (*C. monnieri* vs. natural grass), year (2024 vs. 2025), and their interaction on predator abundance in vegetation strips, corresponding to the hypothesis that *C. monnieri* enhances natural enemy recruitment. One-way ANOVA was used to compare differences among maize field treatments (CM, CM+YC, CM+PL, CM+Imi, and NG) in predator abundance, pest abundance, Shannon diversity index, species richness, control efficacy, and relative humidity, in order to evaluate the effects of endophytic fungi and their integration with *C. monnieri* on biological control performance (Hypotheses 2 and 3). Following significant ANOVA results, pairwise comparisons among means were performed using Fisher’s least significant difference (LSD) test at *p* < 0.05. Considering that measurements were collected from the same plots across two experimental years, additional linear mixed models were conducted to account for potential temporal dependence. Treatment and year were considered fixed factors, whereas plot identity was included as a random factor.

## 3. Results

### 3.1. Differences in Predator Density and Diversity Between C. monnieri and Natural Grass

**Figure 2:**
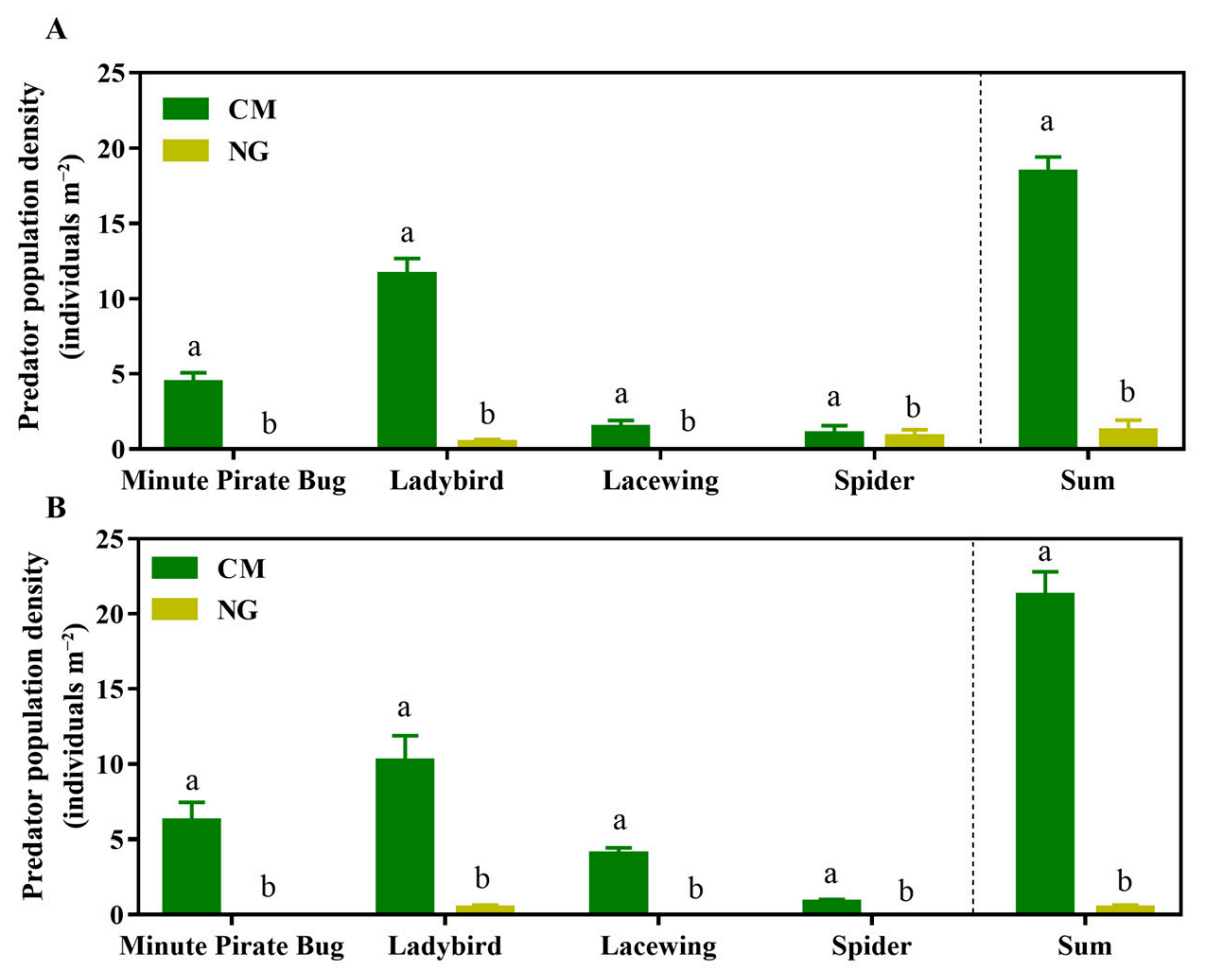
Predator population density on *C. monnieri* (CM) and natural grass (NG) from 2024 to 2025. (**A**) 2024 and (**B**) 2025. Bars represent mean ± standard error (SE). Different lowercase letters above bars indicate significant differences between CM and NG within the same predator group (*p* < 0.05). “Sum” represents the total predator density across all groups. The dashed line separates individual predator groups from the total predator density (“Sum”).

Two-way ANOVA showed that vegetation type significantly affected predator population density (*F* = 349.10, *p* < 0.001). Predator densities were consistently higher on *C. monnieri* (CM) planted at field edges compared to natural grass (NG) (*p* < 0.05; Figure 2). During 2024–2025, minute pirate bugs and lacewings were absent from NG, and spiders were not observed on NG in 2025. Ladybird densities on CM were 29.0- and 25.5-fold higher than those on NG in 2024 and 2025, respectively. The total predator density on CM was 15.33- and 53.0-fold higher than that on NG in 2024 and 2025, respectively. These results indicate that *C. monnieri* supports substantially greater predator population density than natural grass across both years.

**Figure 3:**
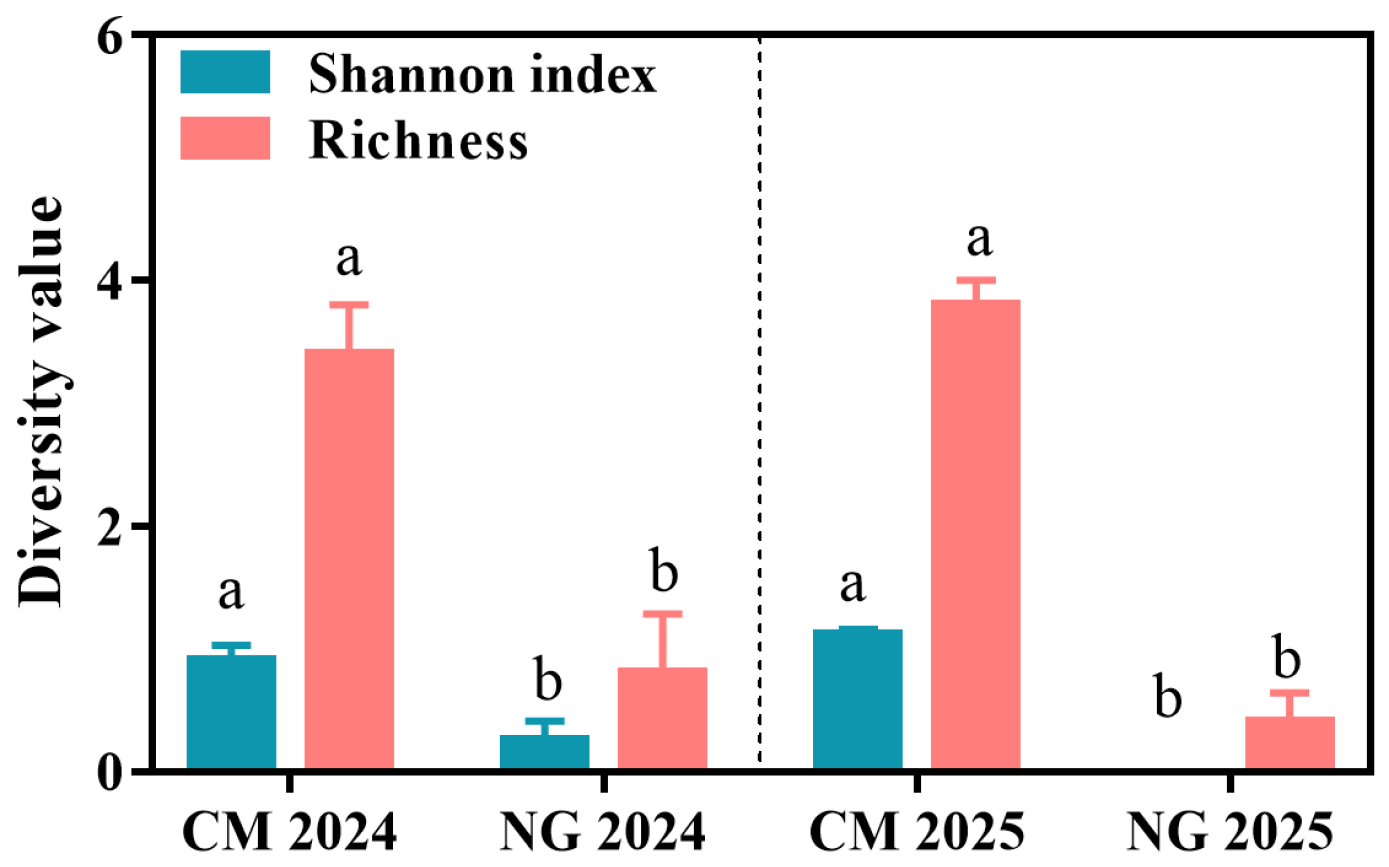
Shannon diversity index and species richness of predator communities on *C. monnieri* (CM) and natural grass (NG) in 2024 and 2025. Different lowercase letters indicate significant differences among treatments (*p* < 0.05). The dashed line separates data from the two sampling years.

Predator community diversity was also significantly improved by *C. monnieri* (Figure 3). Both the Shannon diversity index and species richness were significantly higher in *C. monnieri* plots than those in NG plots in 2024 and 2025 (*p* < 0.05). These results suggest that field-margin planting not only increases predator abundance but also promotes a more diverse predator community.

### 3.2. Effects of C. monnieri Planting and Endophyte Coating on Predator Density and Diversity in Maize Fields

**Figure 4:**
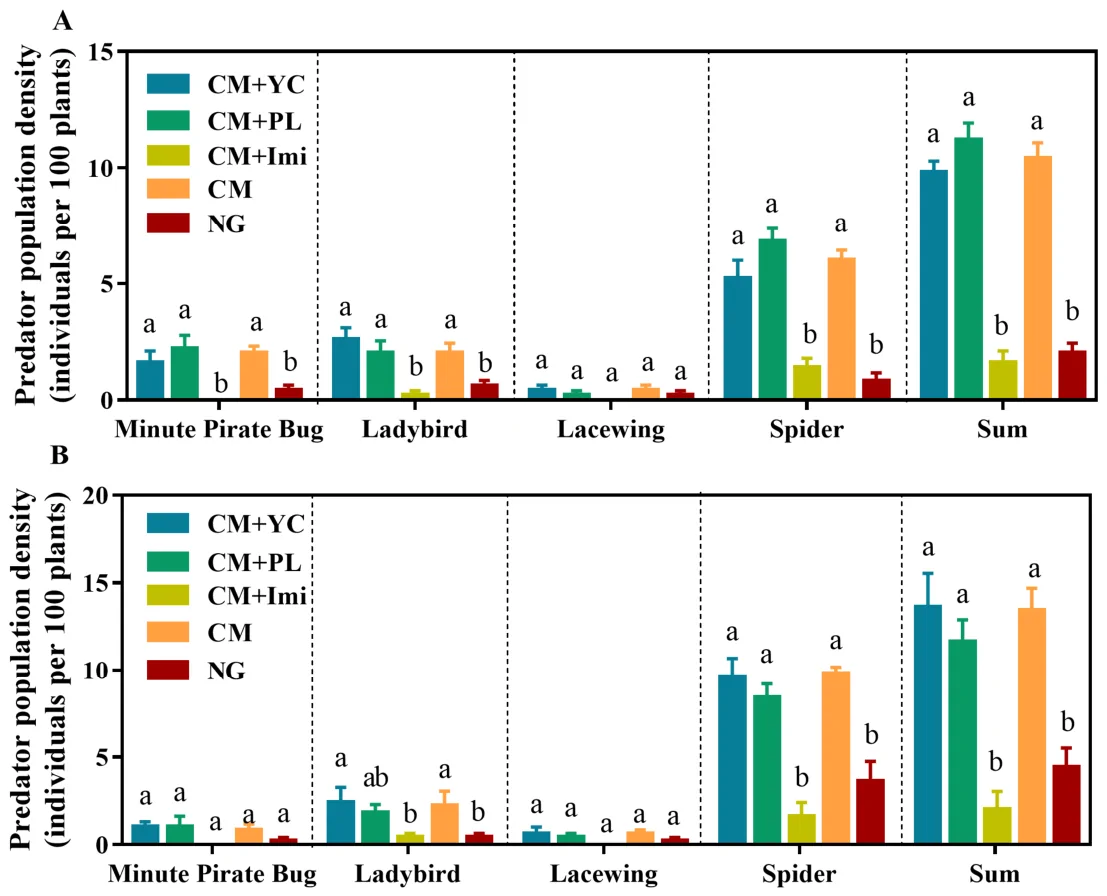
Predator population density during the maize seedling stage in 2024 (**A**) and 2025 (**B**) under *C. monnieri* alone (CM), CM with endophytic fungus YC seed coating (CM+YC), CM with endophytic fungus PL seed coating (CM+PL), CM with imidacloprid seed coating (CM+Imi), and natural grass (NG). Different lowercase letters above bars indicate significant differences among treatments within each predator group (*p* < 0.05). The dashed lines separate predator groups, and “Sum” represents the total predator density across all groups. The same as in the following figures.

Predator population density varied among treatments of *C. monnieri* planting and endophyte coating during the maize seedling stage in 2024 and 2025 (Figure 4). Under conditions where *C. monnieri* was planted at the field edges, no significant differences were detected in the population densities of minute pirate bugs, ladybirds, lacewings, spiders, or the total predator community between maize plots with endophytic fungus–coated seeds (CM+YC and CM+PL) and those with uncoated seeds (CM) in both 2024 and 2025 (*p* > 0.05; Figure 4). In contrast, predator population densities in the CM, CM+YC, and CM+PL treatments were consistently higher than those in plots with imidacloprid-coated maize seeds (CM+Imi) and natural grass (NG). In 2024, the densities of minute pirate bugs, ladybirds, spiders, and the total predator community were significantly greater in CM, CM+YC, and CM+PL treatments compared with those in CM+Imi and NG treatments (*p* < 0.05). A similar pattern was observed in 2025, with ladybird, spider, and total predator densities remaining significantly higher under CM, CM+YC, and CM+PL than those under CM+Imi and NG (*p* < 0.05).

**Figure 5:**
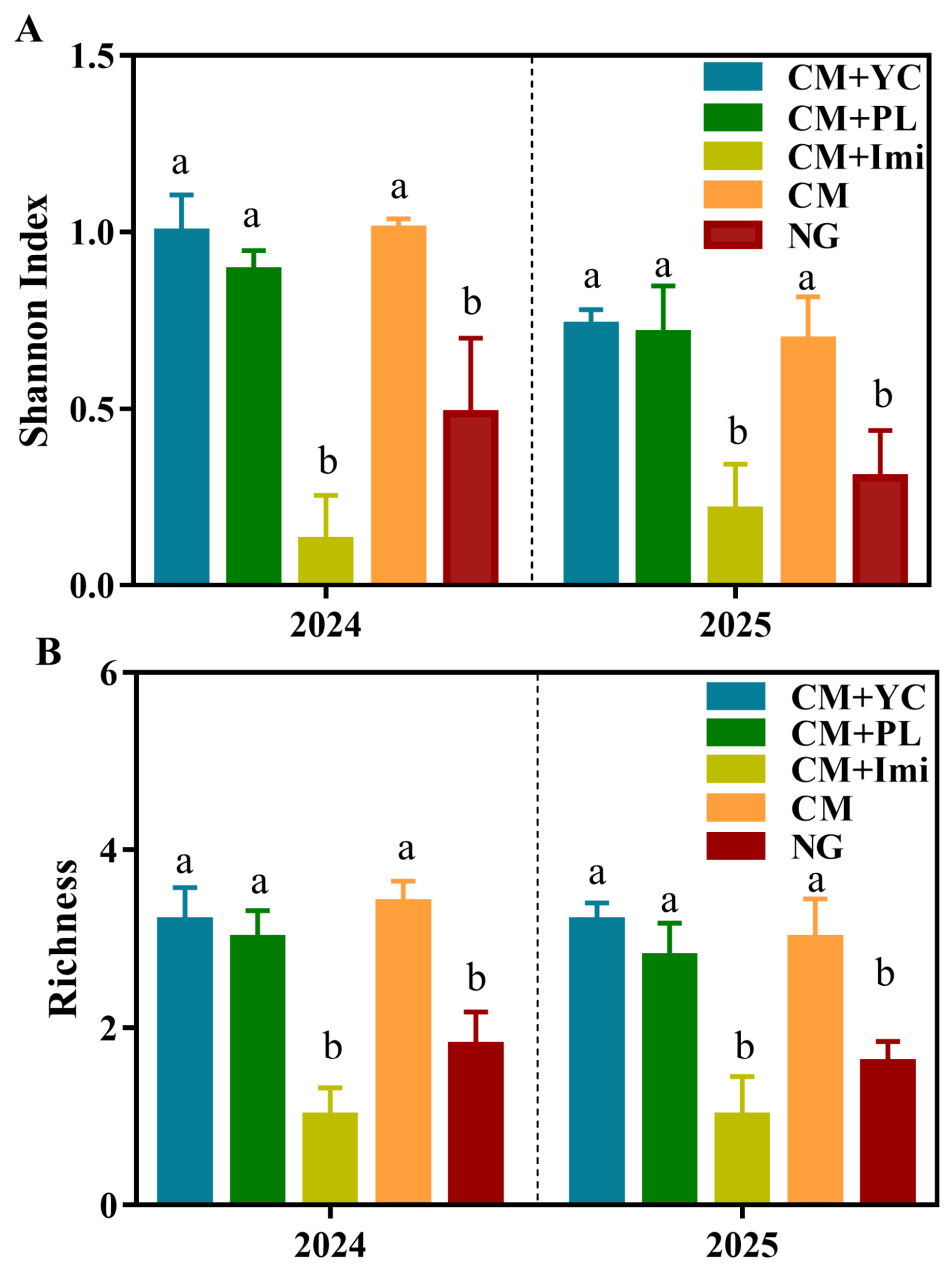
Shannon diversity index (**A**) and species richness (**B**) of predator communities in maize fields in 2024 and 2025 under *C. monnieri* alone (CM), CM with endophytic fungus YC seed coating (CM+YC), CM with endophytic fungus PL seed coating (CM+PL), CM with imidacloprid seed coating (CM+Imi), and natural grass (NG). Different lowercase letters above bars indicate significant differences among treatments within the same year (*p* < 0.05). The dashed vertical line separates data from 2024 and 2025.

The Shannon diversity index and species richness of predator communities were significantly lower in the CM+Imi and NG treatments than those in the other *C. monnieri*-based treatments (CM, CM+YC, and CM+PL) in both 2024 and 2025 (*p* < 0.05; Figure 5). No significant differences were observed among the CM, CM+YC, and CM+PL treatments for either the Shannon index or species richness (*p* > 0.05).

Additional linear mixed model analyses considering plot identity as a random effect confirmed the significant effects of treatments on predator abundance, Shannon diversity index and species richness, consistent with the results obtained from ANOVA (Table S1).

### 3.3. Evidence for Spillover of Predators from C. monnieri to Maize Fields

The detection rate of *C. monnieri* DNA in ladybird gut contents was consistently high across all sampling distances, reaching 100% at 10 m from the *C. monnieri* plots (Table S2). Although the detection rate gradually declined with increasing distance, *C. monnieri* DNA remained detectable in 93.33%, 86.67%, and 80% of ladybirds collected at 20, 30, and 40 m, respectively. However, no significant differences were observed among the four distances (*p* > 0.05). In contrast, *C. monnieri* DNA was not detected in any ladybirds collected from areas adjacent to natural grass plots, with a detection rate of 0% at all sampling distances.

Microclimatic measurements conducted during the maize seedling stage (09:25–16:05) showed that the relative humidity on maize plants was consistently higher than that on *C. monnieri* plants (+37.94%; *p* < 0.05; Figure S1).

### 3.4. Enhanced Biological Control of Maize Pests via C. monnieri Planting and Endophyte Coating

**Figure 6:**
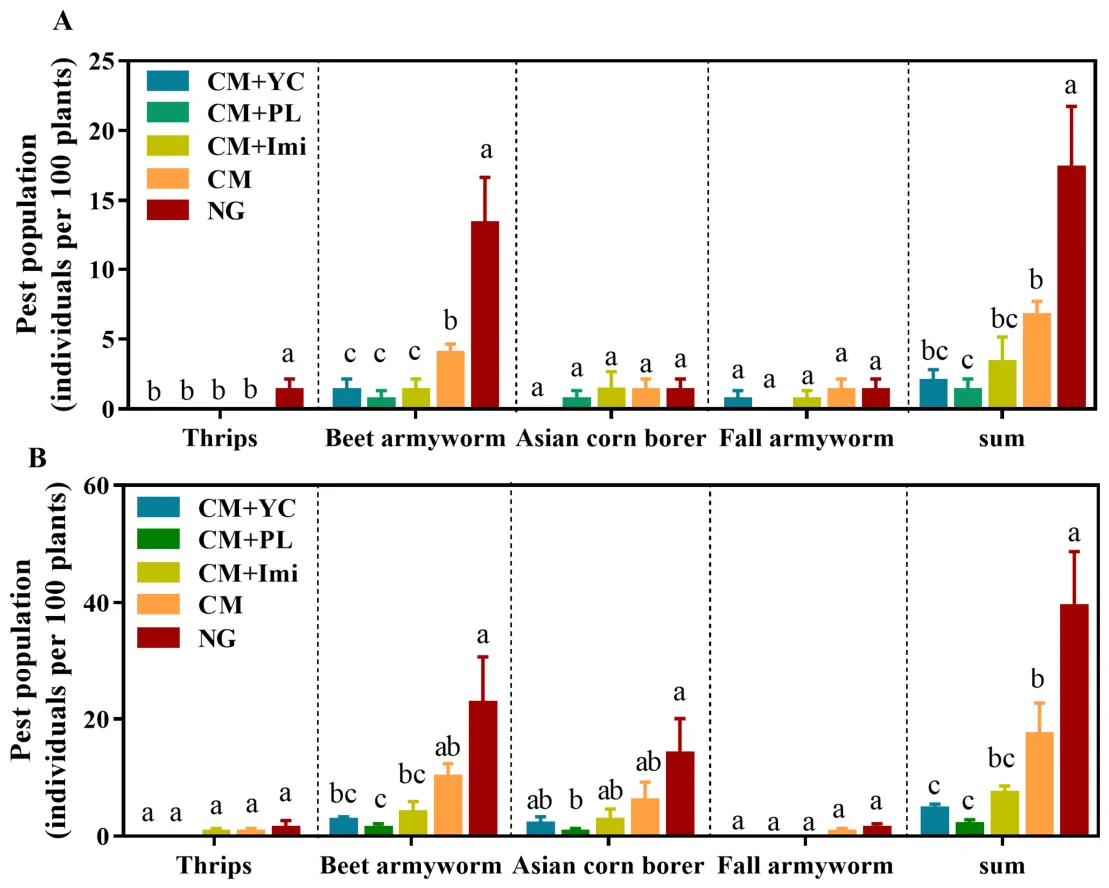
Pest populations recorded during the maize seedling stage in 2024 (**A**) and 2025 (**B**) under *C. monnieri* alone (CM), CM with endophytic fungus YC seed coating (CM+YC), CM with endophytic fungus PL seed coating (CM+PL), CM with imidacloprid seed coating (CM+Imi), and natural grass (NG) in maize fields. Different lowercase letters above bars indicate significant differences among treatments within each pest species or total abundance (sum) (*p* < 0.05). The dashed lines separate different pest species, and “Sum” represents the total pest abundance across all recorded species.

Across both 2024 and 2025, planting *C. monnieri* at field edges combined with endophytic fungus seed coating treatments significantly reduced pest populations in maize fields compared with the NG treatment (*p* < 0.05; Figure 6). No thrips were recorded in maize plots with *C. monnieri* combined with endophytic fungus seed coatings (CM+YC and CM+PL) in either year. In addition, fall armyworm was not detected in CM+YC, CM+PL and CM treatments in 2025. For beet armyworm, in 2024, all *C. monnieri*-based treatments (CM+YC, CM+PL, CM+Imi, and CM) had significantly lower population densities than the NG treatment (*p* < 0.05). Among these, CM+YC and CM+PL did not differ significantly from CM+Imi but were significantly lower than CM (*p* < 0.05). In 2025, no significant difference was observed between CM and NG; however, the CM+PL treatment had a significantly lower beet armyworm population than CM (*p* < 0.05). Regarding total pest abundance, all *C. monnieri*-based treatments consistently showed significantly lower values than the NG treatment in both years ((−55.93%)–(−94.92%); *p* < 0.05). In 2024, the CM+PL treatment had significantly lower total pest abundance than CM (−80%), while not differing significantly from CM+YC and CM+Imi (*p* < 0.05). In 2025, total pest abundance in CM+YC (−73.08%) and CM+PL (−88.46%) treatments was significantly lower than in CM, but did not differ significantly from CM+Imi.

**Figure 7:**
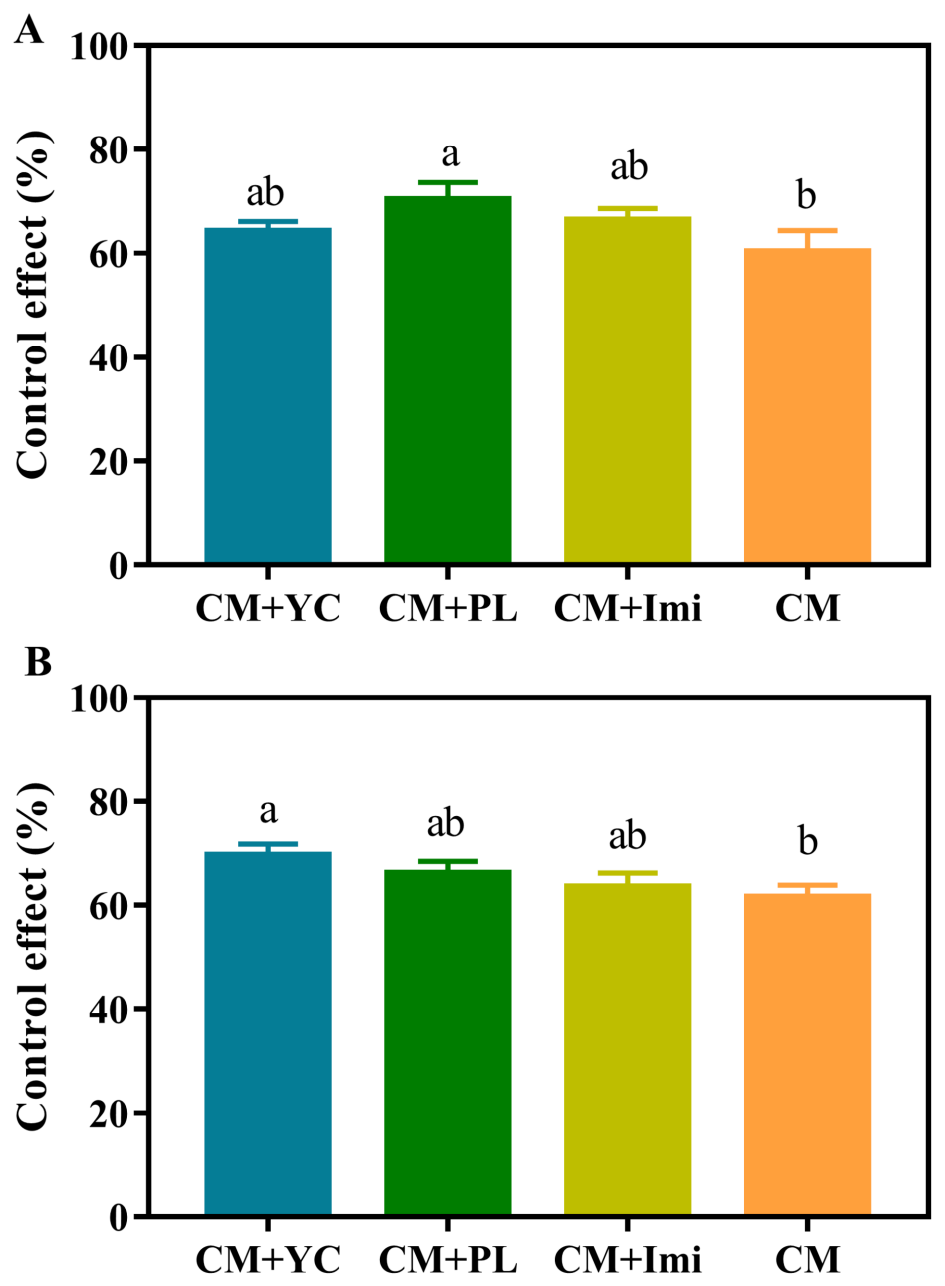
Control effects of *C. monnieri* alone (CM), CM with endophytic fungus YC seed coating (CM+YC), CM with endophytic fungus PL seed coating (CM+PL), CM with imidacloprid seed coating (CM+Imi), and natural grass (NG) on maize pests during the seedling stage in 2024 (**A**) and 2025 (**B**). Different lowercase letters above bars indicate significant differences among treatments (*p* < 0.05).

Planting *C. monnieri* combined with endophytic seed coating significantly enhanced the control efficacy against maize pests (Figure 7). In 2024, CM+PL treatment showed the highest control effect, which was significantly greater than that of CM treatment (+16.74%; *p* < 0.05), but did not differ significantly from CM+YC or CM+Imi treatment (*p* > 0.05; Figure 7A). In 2025, the control effect was highest in CM+YC treatment and significantly higher than that in CM treatment (+13.17%; *p* < 0.05; Figure 7B). The control efficacy of the CM+Imi treatment did not differ significantly from that of the CM+YC or CM+PL treatment (*p* > 0.05). Linear mixed model analyses further confirmed that treatment significantly affected pest populations and control efficacy, and the results were consistent with those obtained from ANOVA analyses (Table S1).

## 4. Discussion

Understanding how multiple ecological processes can be integrated to enhance natural pest regulation is fundamental for developing sustainable crop protection strategies. Habitat diversification through field-margin planting has been widely recognized as an effective approach for conserving natural enemies and promoting top-down control [19,31], while endophytic seed coating represents an emerging plant-mediated strategy for improving host resistance against insect herbivores [27,33]. Nevertheless, the combined ecological consequences of habitat-mediated and endophyte-associated pest suppression, particularly during the vulnerable stage when pest populations become established, remain largely unexplored. Here, we bridge this knowledge gap by combining *C. monnieri* field-margin planting with endophytic seed coating to evaluate their individual and complementary contributions to predator enhancement, pest suppression, and biological control in maize agroecosystems.

*C. monnieri* has received considerable attention as a functional field-margin plant due to its capacity to conserve beneficial arthropods. However, previous studies have largely focused on *C. monnieri* functions within wheat ecosystems [19,34]. In wheat–maize rotation systems, wheat harvest represents a critical ecological bottleneck, as the rapid removal of crop vegetation results in substantial losses of shelter, alternative resources, and suitable habitats for natural enemies [35,36]. Coincidently, *C. monnieri* is in its flowering period during this transition, potentially providing continuous floral resources and refuge habitats that support predator persistence after wheat harvest and before maize canopy establishment [19]. In this study, we demonstrated that the densities of multiple predator taxa, including ladybirds, lacewings, minute pirate bugs, and spiders, were consistently higher in *C. monnieri* habitats, accompanied by increased predator species richness and Shannon diversity. These findings indicate that *C. monnieri* provides a more suitable habitat for supporting diverse predator assemblages than unmanaged natural grass vegetation. The enhanced predator recruitment in *C. monnieri* habitats may be associated with its flowering characteristics and vegetation structure, which potentially provide additional trophic resources and refuge habitats for natural enemies [31,36]. However, because the *C. monnieri* and natural grass habitats were established at different positions within the same experimental field, vegetation effects may partially interact with spatial variation. Although buffer zones and spatial separation were applied to minimize potential interference, future experiments incorporating multiple independently replicated vegetation patches across different locations are needed to further disentangle habitat effects from spatial effects.

Building on the role of *C. monnieri* as a resource-rich refuge habitat, we further investigated whether predators recruited by *C. monnieri* could disperse into adjacent maize fields and promote the establishment and persistence of predator populations within maize fields. Our results demonstrate that maize fields associated with *C. monnieri* consistently supported higher predator abundance, diversity, and species richness than imidacloprid-treated and natural grass treatments, indicating that *C. monnieri* planting can improve the stability of natural enemy communities by providing complementary trophic resources and suitable habitats. Importantly, gut-content analysis detected *C. monnieri* DNA in ladybirds collected from maize fields 10–40 m away from the *C. monnieri* plots, with consistently high detection rates (80–100%) across all distances. These results provide direct molecular evidence of resource utilization and predator spillover from the functional plant habitat into the surrounding crop habitat. Moreover, the significantly higher relative humidity observed in maize plants compared with *C. monnieri* habitats (+37.94%, *p* < 0.05) suggests a potential role of microclimatic conditions in facilitating predator retention after dispersal. Vegetation-mediated modification of microclimatic conditions, including increased humidity and reduced environmental stress, has been recognized as an important mechanism promoting the survival and retention of natural enemies in agricultural landscapes [37,38]. Together, these findings reveal a complementary ecological mechanism in which *C. monnieri* functions as a multifunctional resource habitat by attracting predators and providing essential trophic resources and refuge, whereas maize plants offer favorable microhabitats that facilitate predator establishment and persistence within the crop habitat. Moreover, endophytic fungus-coated maize were compatible with *C. monnieri* planting, maintaining predator abundance and diversity while supporting significantly richer predator communities than imidacloprid-treated plots.

Across two consecutive years, the combined use of *C. monnieri* planting and endophytic fungus-coated seeds consistently achieved comparable pest suppression. Notably, thrips were not detected in maize plots under this combined treatment. This integration provides a complementary pest management system: *C. monnieri* enhances predator recruitment and persistence [17,18], whereas endophytic seed coating provides a complementary bottom-up component associated with reduced pest abundance [39,40,41]. Compared with imidacloprid treatment, this system delivers similar pest control while maintaining higher predator abundance and diversity, underscoring the advantage of integrating functional plants with microbial-based strategies to enhance biological control and minimize the ecological costs associated with chemical insecticides.

Although our two-year field experiments provide evidence that integrating *C. monnieri* planting with endophytic fungus-coated maize seeds can enhance biological control during the maize seedling stage, several limitations should be considered. First, this study was conducted at a single experimental site using one maize hybrid (Zhengdan 958), and the effectiveness of this strategy may vary with environmental conditions, landscape context, soil properties, and crop genotypes. Therefore, multi-location trials involving diverse maize varieties are needed to further evaluate the general applicability and stability of this approach. Second, although consistent effects were observed across two consecutive growing seasons (2024–2025), longer-term experiments covering multiple years and crop growth stages are required to determine whether these ecological benefits persist over time and ultimately translate into improvements in crop productivity, including biomass accumulation, grain filling, and yield. Third, while previous studies have demonstrated the insect-resistance potential of the YC strain, endophytic colonization and the associated physiological mechanisms were not directly investigated in this field study. Future research integrating fungal colonization analysis, plant defense responses, and metabolomic or transcriptomic approaches will help elucidate the mechanisms underlying endophyte-mediated pest suppression. Finally, our assessment focused mainly on dominant aboveground predatory arthropods, including ladybirds, minute pirate bugs, lacewings, and spiders. Future studies incorporating parasitoids, pollinators, and soil arthropod communities will provide a more comprehensive evaluation of the ecological effects of this integrated pest management strategy.

## 5. Conclusions

Our study demonstrates that integrating *C. monnieri* field-margin planting with endophytic fungus-coated maize seeds provides an effective and ecologically sustainable strategy for pest management. *C. monnieri* enhanced predator abundance, diversity, and spillover into adjacent maize fields, whereas endophytic seed coating provided complementary pest suppression. The combined system achieved pest control comparable to imidacloprid while conserving a more diverse and abundant natural enemy community. These findings demonstrate the compatibility and complementary effects of habitat management and endophyte-based pest management, although the physiological mechanisms underlying the endophyte-associated pest suppression remain to be resolved.

## Data Availability

Due to the confidentiality agreement, this dataset cannot be fully disclosed temporarily. However, if there is a research need, the researchers can contact the author via email to obtain it.

## Supplementary Materials

The following supporting information can be downloaded at: https://www.mdpi.com/article/10.3390/insects17080872/s1. Figure S1: Microclimatic conditions associated with *Cnidium monnieri* and maize plants during the maize seedling stage; Table S1: Results of linear mixed models for predator population density, Shannon index, richness, pest population, and control effect; Table S2: Detection rate of Cnidium monnieri DNA in the gut contents of ladybirds collected from maize fields at different distances from C. monnieri or natural grass.

## References

[1] Ma C.S., Wang B.X., Wang X.J., Lin Q.C., Zhang W., Yang X.F., van Baaren J., Bebber D.P., Eigenbrode S.D., Zalucki M.P. (2025). Crop pest responses to global changes in climate and land management. Nat. Rev. Earth Environ..

[2] Skendžić S., Zovko M., Živković I.P., Lešić V., Lemić D. (2021). The Impact of Climate Change on Agricultural Insect Pests. Insects.

[3] Deutsch C.A., Tewksbury J.J., Tigchelaar M., Battisti D.S., Merrill S.C., Huey R.B., Naylor R.L. (2018). Increase in crop losses to insect pests in a warming climate. Science.

[4] Begg G.S., Cook S.M., Dye R., Ferrante M., Franck P., Lavigne C., Lövei G.L., Mansion-Vaquie A., Pell J.K., Petit S. (2017). A functional overview of conservation biological control. Crop Prot..

[5] Landis D.A., Gardiner M.M., van der Werf W., Swinton S.M. (2008). Increasing corn for biofuel production reduces biocontrol services in agricultural landscapes. Proc. Natl. Acad. Sci. USA.

[6] Tscharntke T., Klein A.M., Kruess A., Steffan-Dewenter I., Thies C. (2005). Landscape perspectives on agricultural intensification and biodiversity—Ecosystem service management. Ecol. Lett..

[7] Seimandi-Corda G., MacLaren C., Tougeron K., Forkman J., Hood J., Mead A., Dixon A., Cook S.M. (2026). Implementation of practices shapes the effectiveness of agricultural diversification for arthropod related ecosystem services: A meta-analysis. Agron. Sustain. Dev..

[8] Martin E.A., Dainese M., Clough Y., Báldi A., Bommarco R., Gagic V., Garratt M.P.D., Holzschuh A., Kleijn D., Kovács-Hostyánszki A. (2019). The interplay of landscape composition and configuration: New pathways to manage functional biodiversity and ecosystem services across Europe. Ecol. Lett..

[9] Lenné J., Wood D. (2024). Crop Diversity in Agroecosystems for Pest Management and Food Production. Plants.

[10] Puliga G.A., Sprangers T., Huiting H.F., Dauber J. (2024). Management practices influence biocontrol potential of generalist predators in maize cropping systems. Entomol. Exp. Appl..

[11] Wei Q., Zhang X., Yang F., Duan S., Fan Z., Nie P., Chen Z., Feng J. (2025). Future Range Shifts in Major Maize Insect Pests Suggest Their Increasing Impacts on Global Maize Production. Insects.

[12] Wang M., Zhang F., Su Y., Hui C., Ge F., Zhang Y., Ouyang F. (2026). Intercropping cotton with maize enhances biological control of aphids by the predatory lacewing *Chrysoperla sinica*. Entomol. Gen..

[13] Holland J.M., Bianchi F.J.J., Entling M.H., Moonen A.C., Smith B.M., Jeanneret P. (2016). Structure, function and management of semi-natural habitats for conservation biological control: A review of European studies. Pest Manag. Sci..

[14] Obanyi J.N., Ogendo J.O., Mulwa R.M.S., Nyaanga J.G., Cheruiyot E.K., Bett P.K., Belmain S.R., Arnold S.E.J., Nash-Woolley V.C., Stevenson P.C. (2024). Flowering margins support natural enemies between cropping seasons. Front. Agron..

[15] Colazza S., Peri E., Cusumano A. (2023). Chemical Ecology of Floral Resources in Conservation Biological Control. Annu. Rev. Entomol..

[16] Albrecht M., Kleijn D., Williams N.M., Tschumi M., Blaauw B.R., Bommarco R., Campbell A.J., Dainese M., Drummond F.A., Entling M.H. (2020). The effectiveness of flower strips and hedgerows on pest control, pollination services and crop yield: A quantitative synthesis. Ecol. Lett..

[17] Li Z., Chang C., Yuan Y., Zhang X., Ge F. (2023). Functional plant, *Cnidium monnieri*, facilitates the conservation and the biocontrol performance of natural enemies. Innov. Geosci..

[18] Su W., Ouyang F., Li Z., Yuan Y., Yang Q., Ge F. (2023). *Cnidium monnieri* (L.) Cusson Flower as a Supplementary Food Promoting the Development and Reproduction of Ladybeetles *Harmonia axyridis* (Pallas) (Coleoptera: Coccinellidae). Plants.

[19] Liang X., Zhang X., Zhao C., Yan L., Zhang X., Chang C., Jiang X., Li Z., Ge F. (2025). Effects of *Cnidium monnieri* planting on the conservation of natural enemy ladybirds and their aphid control effects in fields in wheat-maize rotation cropping system. Acta Entomol. Sin..

[20] Chaudhary P., Agri U., Chaudhary A., Kumar A., Kumar G. (2022). Endophytes and their potential in biotic stress management and crop production. Front. Microbiol..

[21] Panwar N., Szczepaniec A. (2024). Endophytic entomopathogenic fungi as biological control agents of insect pests. Pest Manag. Sci..

[22] Vieira M.E.O., Nunes V.V., Calazans C.C., Silva-Mann R. (2024). Unlocking plant defenses: Harnessing the power of beneficial microorganisms for induced systemic resistance in vegetables—A systematic review. Biol. Control.

[23] Tao J., Gu M., Yu S., Shi J., Cheng L., Jin J., Lu P., Zhang J., Li H., Cao P. (2024). The beneficial endophytic microbes enhanced tobacco defense system to resist bacterial wilt disease. Chem. Biol. Technol. Agric..

[24] Pandey P., Tripathi A., Dwivedi S., Lal K., Jhang T. (2023). Deciphering the mechanisms, hormonal signaling, and potential applications of endophytic microbes to mediate stress tolerance in medicinal plants. Front. Plant Sci..

[25] Darsouei R., Karimi J., Stelinski L.L. (2024). Endophytic colonization of sugar beet by *Beauveria varroae* and *Beauveria bassiana* reduces performance and host preference in army worm, *Spodoptera littoralis*. Crop Prot..

[26] Paravar A., Piri R., Balouchi H., Ma Y. (2023). Microbial seed coating: An attractive tool for sustainable agriculture. Biotechnol. Rep..

[27] Song Y., Cui H., Guo W., Lara S., Lv S., Li L., Yu Y., Men X. (2024). Endophytic fungi improved wheat resistance to *Rhopalosiphum padi* by decreasing its feeding efficiency and population fitness. Ecotoxicol. Environ. Saf..

[28] Ahsan S.M., Hoque M.I.-U., Das A.K., Rahman M.M., Mollah M.M.I., Paul N.C., Choi H.W. (2024). Plant–Entomopathogenic Fungi Interaction: Recent Progress and Future Prospects on Endophytism-Mediated Growth Promotion and Biocontrol. Plants.

[29] Sena L., Mica E., Valè G., Vaccino P., Pecchioni N. (2024). Exploring the potential of endophyte-plant interactions for improving crop sustainable yields in a changing climate. Front. Plant Sci..

[30] Sun Y., Men X., Li C., Yu Y., Lü S., Sun T., Ye B., Li L. (2020). Effects of highly insect resistant corn endophytic fungi on growth, reproduction and feeding preference of *Rhopalosiphum maidis*. Chin. J. Biol. Control.

[31] Dong Z., Yang Q., Wyckhuys K.A.G., Ouyang F., Gurr G.M., Han P., González-Chang M., van der Werf W., Men X., Ge F. (2026). Use of functional plants to enhance pest biological control in farming landscapes: Progress and prospects. Entomol. Gen..

[32] Yang Q., Men X., Zhao W., Li C., Zhang Q., Cai Z., Ge F., Ouyang F. (2021). Flower strips as a bridge habitat facilitate the movement of predatory beetles from wheat to maize crops. Pest Manag. Sci..

[33] Das D., Sharma P.L., Paul P., Baruah N.R., Choudhury J., Begum T., Karmakar R., Khan T., Kalita J. (2025). Harnessing endophytes: Innovative strategies for sustainable agricultural practices. Discov. Bact..

[34] Jiang X., Zhao L., Sergers A., Chang C., Zhang X., Ju Q., Ge F. (2025). Herbivore-induced plant volatiles (HIPVs) from companion plant could enhance predator recruitment and biocontrol of cereal aphids. Entomol. Gen..

[35] González E., Bianchi F.J.J.A., Eckerter P.W., Pfaff V., Weiler S., Entling M.H. (2022). Ecological requirements drive the variable responses of wheat pests and natural enemies to the landscape context. J. Appl. Ecol..

[36] Rusch A., Valentin-Morison M., Sarthou J.-P., Roger-Estrade J., Sparks D.L. (2010). Chapter six—Biological control of insect pests in agroecosystems: Effects of crop management, farming systems, and seminatural habitats at the landscape scale: A review. Advances in Agronomy.

[37] Landis D.A., Wratten S.D., Gurr G.M. (2000). Habitat Management to Conserve Natural Enemies of Arthropod Pests in Agriculture. Annu. Rev. Entomol..

[38] Gurr G.M., Wratten S.D., Landis D.A., You M. (2017). Habitat Management to Suppress Pest Populations: Progress and Prospects. Annu. Rev. Entomol..

[39] Qin T., Shi X., Yin J., Qu Y., Deng Y., Wei X., Zhao N., Gao Y., Mace W.J., Ren A. (2025). Fungal endophytes enhanced insect resistance by improving the defenses of *Achnatherum sibiricum* before locust feeding. Pest Manag. Sci..

[40] Zhou W., Starr J.L., Krumm J.L., Sword G.A. (2016). The fungal endophyte *Chaetomium globosum* negatively affects both above- and belowground herbivores in cotton. FEMS Microbiol. Ecol..

[41] Lopez D.C., Sword G.A. (2015). The endophytic fungal entomopathogens *Beauveria bassiana* and *Purpureocillium lilacinum* enhance the growth of cultivated cotton (*Gossypium hirsutum*) and negatively affect survival of the cotton bollworm (*Helicoverpa zea*). Biol. Control.

