# Supplementary material for: Enhancing Biological Control of Maize Seedling-Stage Pests Through Combined Application of Cnidium monnieri and Endophytes
Source: Insects. 2026 Aug 21;17(8):872. doi: 10.3390/insects17080872 (PMC13513143; doi:10.3390/insects17080872)
Supplement: Supplementary file 1 [file insects-17-00872-s001.zip › insects-4469400-supplementary.pdf]

Supporting Information

Table S1. Results of linear mixed models for predator population density, Shannon index, richness, pest population, and control effect.

Response variable	Effect	*F*	*p*
Predator population density	Treatment	20.42	<0.001
	Year	6.38	0.03
	Treatment × Year	0.43	0.79
Shannon index	Treatment	17.88	<0.001
	Year	3.43	0.08
	Treatment × Year	0.98	0.45
Richness	Treatment	12.00	<0.001
	Year	0.06	0.82
	Treatment × Year	0.29	0.88
Pest population	Treatment	12.15	<0.001
	Year	6.91	0.009
	Treatment × Year	1.49	0.2
Control effect	Treatment	3.94	0.03
	Year	0.13	0.72
	Treatment × Year	2.62	0.09

Table S2. Detection rate of *Cnidium monnieri* DNA in the gut contents of ladybirds collected from maize fields at different distances from *C. monnieri* or natural grass.

Treatments	Detection rate (%)			
	10 m	20 m	30 m	40 m
C. monnieri	100 a	93.33 a	86.67 a	80 a
Natural grass	0	0	0	0

Note: The same lowercase letters indicate no significant differences (p > 0.05).

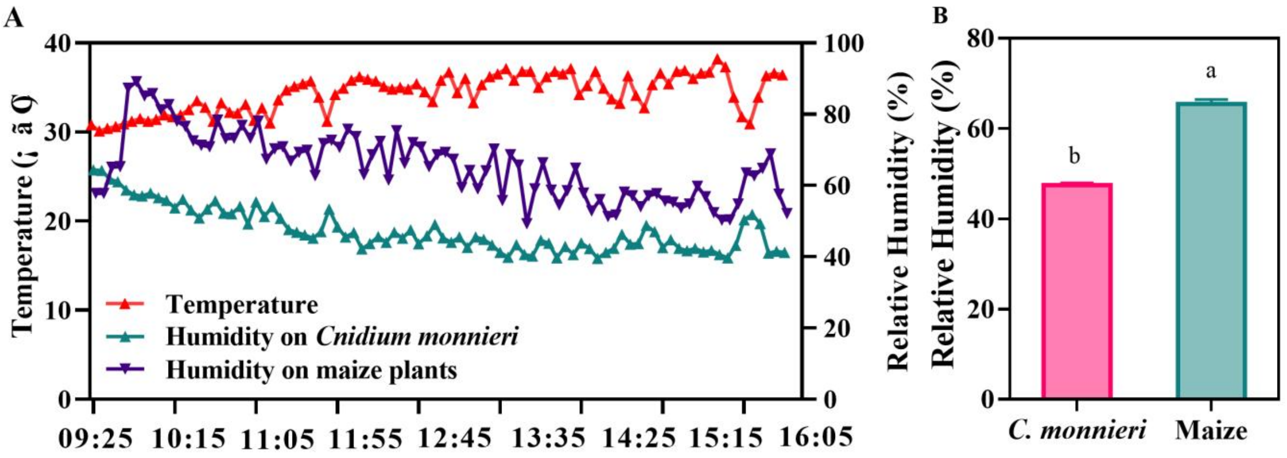

Figure S1. Microclimatic conditions associated with *Cnidium monnieri* and maize plants during the maize seedling stage. (A) Temporal variation in air temperature (°C) and relative humidity (%) measured near *C. monnieri* and maize plants from 09:25 to 16:05. (B) Mean relative humidity recorded in the vicinity of *C. monnieri* and maize plants. Bars represent mean ± standard error (SE).
